# Percutaneous Repair of Ventricular Ruptures

**DOI:** 10.1016/j.jaccas.2019.11.088

**Published:** 2020-03

**Authors:** Rong Bing, Miles W.H. Behan, Renzo Pessotto, Nicholas L.M. Cruden, David B. Northridge

**Affiliations:** a Edinburgh Heart Centre, Royal Infirmary of Edinburgh, Edinburgh, United Kingdom; b British Heart Foundation Centre for Cardiovascular Science, University of Edinburgh, Edinburgh, United Kingdom

**Keywords:** apical cannulation, CT, computed tomography, left ventricular pseudoaneurysm, ventricular septal defect, CT, computed tomography, IABP, intra-aortic balloon pump, LV, left ventricular, MDT, multidisciplinary team, RV, right ventricular, TEE, transesophageal echocardiography, TTE, transthoracic echocardiogram, VSD, ventricular septal defect

## Abstract

Acquired ventricular wall ruptures can be life-threatening. Depending on the pathological features and anatomy, surgical repair can be technically challenging and may be associated with high morbidity and mortality. We present 3 successful percutaneous repairs of different ruptures that used a variety of techniques. (**Level of Difficulty: Advanced.**)

Cardiac rupture is a potentially life-threatening complication of myocardial infarction. Ventricular septal defects (VSDs) are the most common form, occurring in <0.5% of myocardial infarctions, and they are associated with a high mortality rate (1). Free wall ruptures can also occur, leading to cardiac tamponade or pseudoaneurysm formation. Iatrogenic pseudoaneurysms are also described following left ventricular (LV) cannulation (2, 3, 4). The timing and modality of repair of these defects remain controversial, and the small body of available observational data is heavily confounded. Issues to be considered include the patient’s comorbidities and hemodynamics, infarct and rupture location, and the lesion anatomy. Although it has been suggested that a lower operative mortality may be incurred by operating after the immediate post-infarct period, this may reflect selection bias, and in the acutely unstable patient, deterioration secondary to cardiogenic shock is inevitable without intervention or hemodynamic support. Pharmacological support with vasoactive agents to maintain end-organ perfusion comes at the expense of increased myocardial workload. Mechanical support with devices such as the intra-aortic balloon pump (IABP) and the Impella pump (Abiomed, Danvers, Massachusetts) reduce myocardial workload, whereas venoarterial extracorporeal membrane oxygenation offers excellent end-organ perfusion at the expense of increased afterload. However, all these devices require moderate- or large-bore arterial cannulation while the device is in situ, with the attendant risk of complications. Percutaneous transcatheter techniques are much less invasive than surgery and offer the potential for definitive repair, although there are no data comparing these approaches. If a percutaneous technique is to be considered as an option, it is essential to delineate the anatomy of the lesion, in particular the defect size (in 3 planes), surrounding tissue rims, and location in relation to potential routes of percutaneous access that may influence the ease of device delivery. Pre-procedural multimodality imaging can be invaluable in this regard. Access to, and familiarity with, a variety of devices is also required.

Here, we present 3 cases of percutaneous repair achieved by different methods (Table 1, Central Illustration)

**Learning Objectives**

• To recognize the varied clinical presentations of acquired ventricular ruptures and their potential causes.
• To understand the anatomy of these lesions and the importance of multimodality imaging in delineating anatomy and guiding a therapeutic strategy.
• To understand the various clinical factors that must be taken into account by the multidisciplinary heart team in deciding whether to intervene, and if so, by what method..

**Table 1:** Case Summaries

Case #	Lesion	Presentation	Imaging and Access	Device
1	LV pseudoaneurysm, inferobasal	2 months post-inferior MI Asymptomatic	TTE CT Fluoroscopy Femoral arterial access	11-mm Amplatzer Septal OccluderDisc diameters: distal 25 mm, proximal 21 mmWaist: diameter 11 mm, length 4 mm
2	LV pseudoaneurysm, apical	2 months post-transapical TAVI Pulsatile chest wall mass	TTE TEE Fluoroscopy Direct percutaneous access	16-mm Amplatzer PI Muscular VSD OccluderDisc diameters: 26 mmWaist: diameter 16 mm, length 10 mm
3	Biventricular rupture with communicating pseudoaneurysm	Acute post-infarct Early cardiogenic shock	TTE TEE Fluoroscopy Femoral venous and arterial access	14-mm Amplatzer Muscular VSD OccluderDisc diameters: 22 mmWaist: diameter 14 mm, length 7 mm

CT = computed tomography; LV = left ventricular; MI = myocardial infarction; PI = post-infarct; TAVI = transcatheter aortic valve implantation; TEE = transesophageal echocardiography; TTE = transthoracic echocardiography; VSD = ventricular septal defect.

**Central Illustration:**
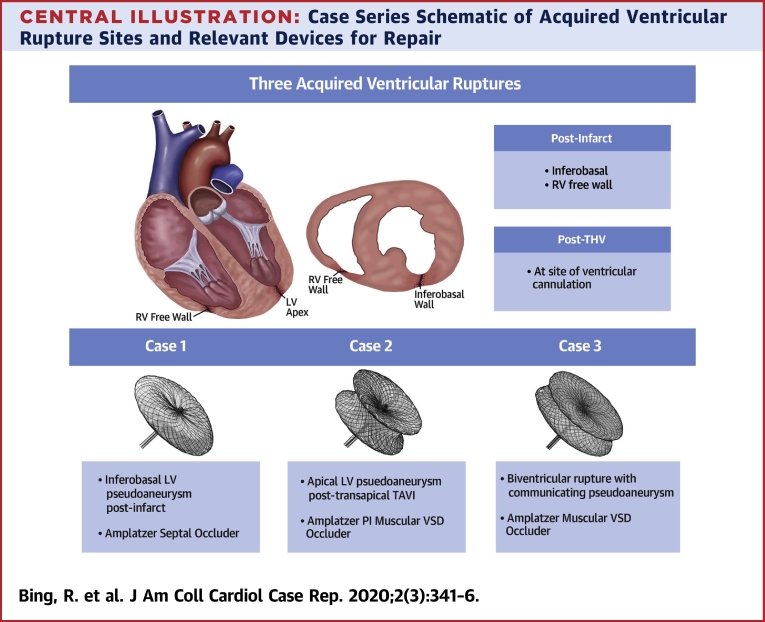
Case Series Schematic of Acquired Ventricular Rupture Sites and Relevant Devices for Repair

## Case 1: Post-Infarct Left Ventricular Pseudoaneurysm

**Figure 1:**
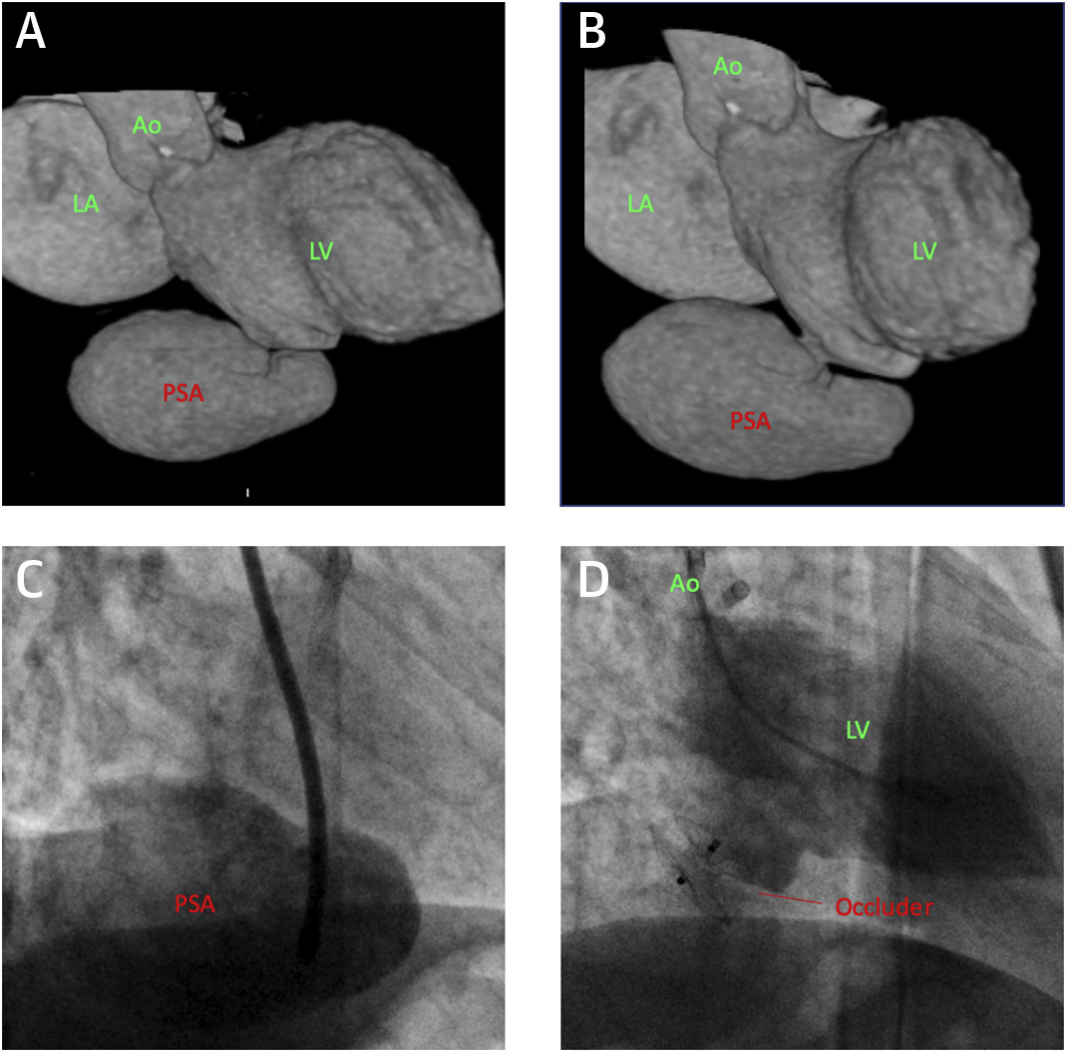
Left Ventricular Inferobasal Pseudoaneurysm **(A and B)** Volume-rendered computed tomography images of the left side of the heart showing the inferobasal pseudoaneurysm (PSA) with a narrow neck. **(C)** The anatomy is confirmed on angiography with contrast injection into the sac. **(D)** Successful occlusion of the pseudoaneurysm. Ao = aorta; LA = left atrium; LV = left ventricle.

A 70-year-old woman underwent primary percutaneous coronary intervention of the right coronary artery for an inferoposterior myocardial infarction. The patient was reviewed 6 months later. She had no symptoms of heart failure. Blood pressure was 140/60 mm Hg, with a heart rate of 80 beats/min and an oxygen saturations of 98%. On examination, a harsh, 3/6 systolic murmur at the left lower sternal edge was noted. Transthoracic echocardiography (TTE) demonstrated a large inferobasal LV pseudoaneurysm, confirmed on computed tomography (CT) (Figures 1A and 1B). The maximum diameter of the pseudoaneurysm neck was 9 mm. The patient was discussed at the multidisciplinary team (MDT) meeting. Although she was asymptomatic, there was a risk of potentially fatal pseudoaneurysm rupture. The delay from the infarct also favored closure, in contrast to repair in the acute peri-infarct period, when tissue rims are often too friable to support a device.

The procedure was performed from the right femoral artery and using local anesthesia with TTE and fluoroscopy. A 5-F Judkins right 4 (JR4) diagnostic catheter was advanced into the left ventricle, and the defect was crossed with a 0.035-inch hydrophilic wire. The JR4 catheter was advanced into the pseudoaneurysm (Figure 1C), and the wire was exchanged for a 0.035-inch Confida wire (Medtronic, Minneapolis, Minnesota). The JR4 catheter was exchanged for a 7-F 90-cm guiding sheath. Because of the defect diameter, as well as the wall thinning of the infarcted area which necessitated a short waist length, an 11-mm Amplatzer Septal Occluder (Abbott, Abbott Park, Illinois) was deployed (Videos 1 and 2). The final angiographic result was satisfactory (Figure 1D). TTE the following day confirmed complete exclusion of the pseudoaneurysm. The patient was discharged home.

This case demonstrates the feasibility of low-risk percutaneous closure of a focal LV rupture. It is important to distinguish the subacute presentation of this patient with acute presentations in the immediate peri-infarct phase. Pre-procedure CT was valuable in delineating the anatomy and allowing selection of an appropriate device. Finally, pre-procedural imaging and favorable apical TTE windows allowed for a safe and efficient procedure using local anesthesia.

## Case 2: Iatrogenic Apical Pseudoaneurysm

An 85-year-old woman with bioprosthetic aortic and mitral valve replacements (23-mm and 29-mm Carpentier-Edwards [Edwards Lifesciences, Irvine, California], respectively) presented with heart failure secondary to severe transvalvular mitral regurgitation. The patient underwent transapical valve-in-valve transcatheter mitral valve implantation with a 29-mm Sapien 3 valve (Edwards Lifesciences). The LV cannulation site was closed with 2 pledgeted pursestring sutures.

**Figure 2:**
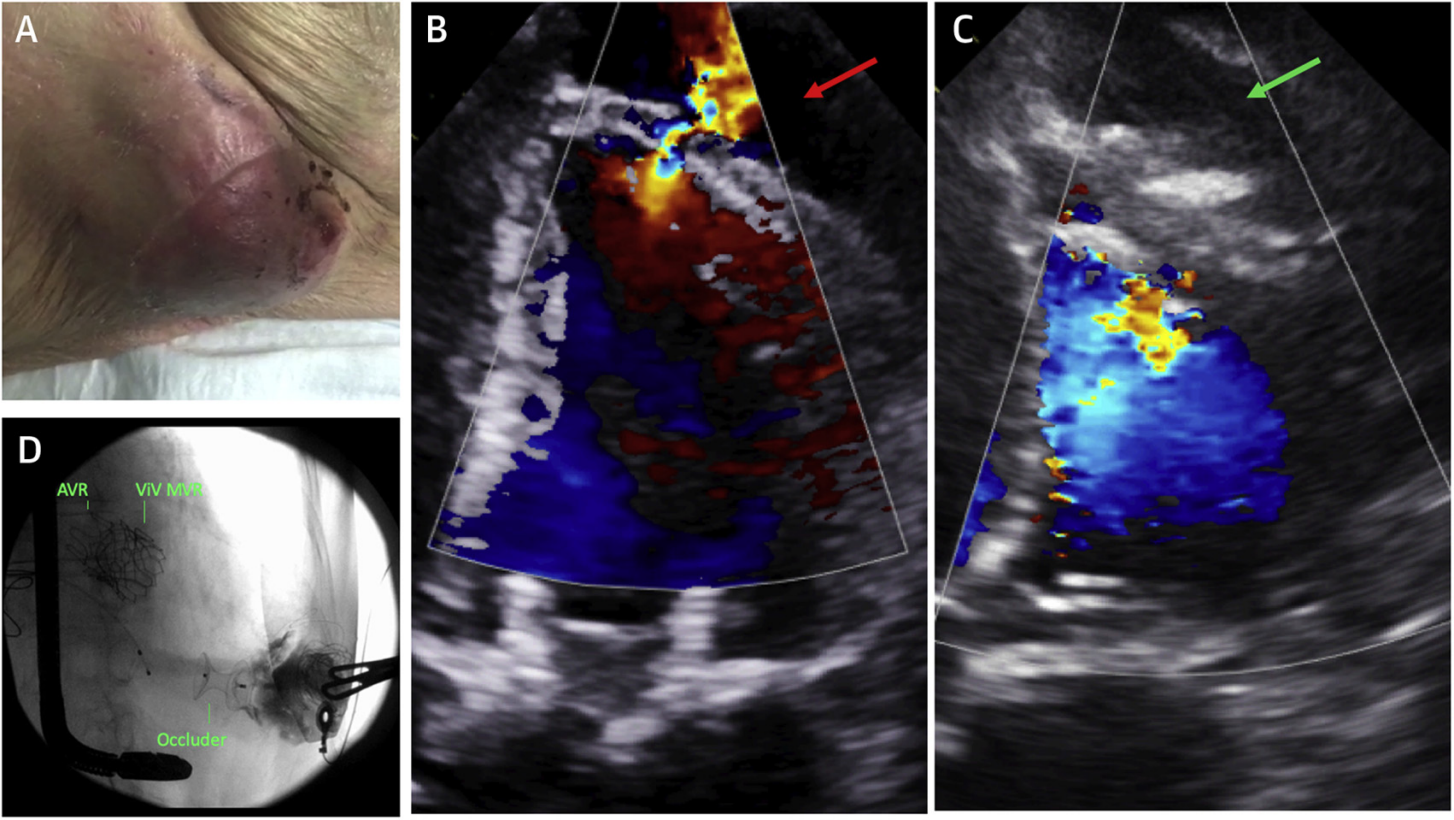
Left Ventricular Apical Pseudoaneurysm **(A)** The apical mass (pulsatile; see Video 3). **(B and C)** 4-chamber transthoracic echocardiographic views with color Doppler imaging showing the defect before **(red arrow)** and after **(green arrow)** device closure. **(D)** Fluoroscopy shows contrast staining in the excluded pseudoaneurysm with an occluder in place. AVR = aortic valve replacement; MVR = mitral valve replacement; ViV = valve-in-valve.

The patient presented 2 months later with an enlarging, pulsatile chest wall mass (Figure 2A, Video 3). Blood pressure was 95/50 mm Hg, with a heart rate of 90 to 110 beats/min (atrial fibrillation) and oxygen saturations of 93%. Echocardiography demonstrated rupture of the LV cannulation site with bidirectional flow into a large pseudoaneurysm (Figure 2B). The patient was discussed at the MDT meeting. Rupture of the pseudoaneurysm in this scenario would almost certainly be fatal, whereas open repair would be very high risk given the patient’s history.

The patient underwent transcatheter repair by a direct percutaneous approach with TTE, transesophageal echocardiography (TEE), and fluoroscopy. TTE imaging was superior to TEE because of the defect location. The pseudoaneurysm was accessed with an 18-gauge needle and the ventricular defect wired with a 0.035-inch hydrophilic wire. After antegrade passage of the wire through the aortic valve, a 9-F sheath was introduced into the pseudoaneurysm, through which a 16-mm Amplatzer PI Muscular VSD Occluder (Abbott) was deployed (Video 4). Echocardiography showed no flow (Figure 2C). Contrast injection into the pseudoaneurysm demonstrated staining with no negative jet (Figure 2D). The patient was discharged home.

This case demonstrates an uncommon complication of transapical transcatheter valve implantation and the feasibility of direct percutaneous closure. This approach is minimally invasive, facilitates hemostasis throughout the case, avoids the requirement for additional arterial access, and provides excellent support for device delivery with the wire in the descending aorta.

## Case 3: Post-Infarct Biventricular Rupture With Communicating Pseudoaneurysm

A 79-year-old woman with chronic kidney disease was admitted to a district hospital with a completed inferior myocardial infarction. A loud systolic murmur was noted. A presumed VSD was identified on TTE. The patient was pain-free but hypotensive and was transferred to our center for further management 2 days after her initial presentation. On arrival, blood pressure was 100/60 mm Hg, with a heart rate of 100 beats/min and an oxygen saturations of 99%. Coronary angiography confirmed an occluded dominant right coronary artery. Repeat TTE demonstrated an inferior LV wall defect with left-to-right flow. The margins were difficult to define but appeared to be contiguous with the septum. An attempt was made to temporize and allow healing of the tissue rim, but heart failure developed and an IABP was inserted 1 week later. Despite this the patient’s condition deteriorated. Additional mechanical support for several weeks as a bridge to therapy was not felt to be feasible. The anatomy was suboptimally defined, but contrast-enhanced CT was not performed because of the patient’s worsening renal function. Surgery was believed to be too high risk in light of the hemodynamics and ambiguous anatomy.

**Figure 3:**
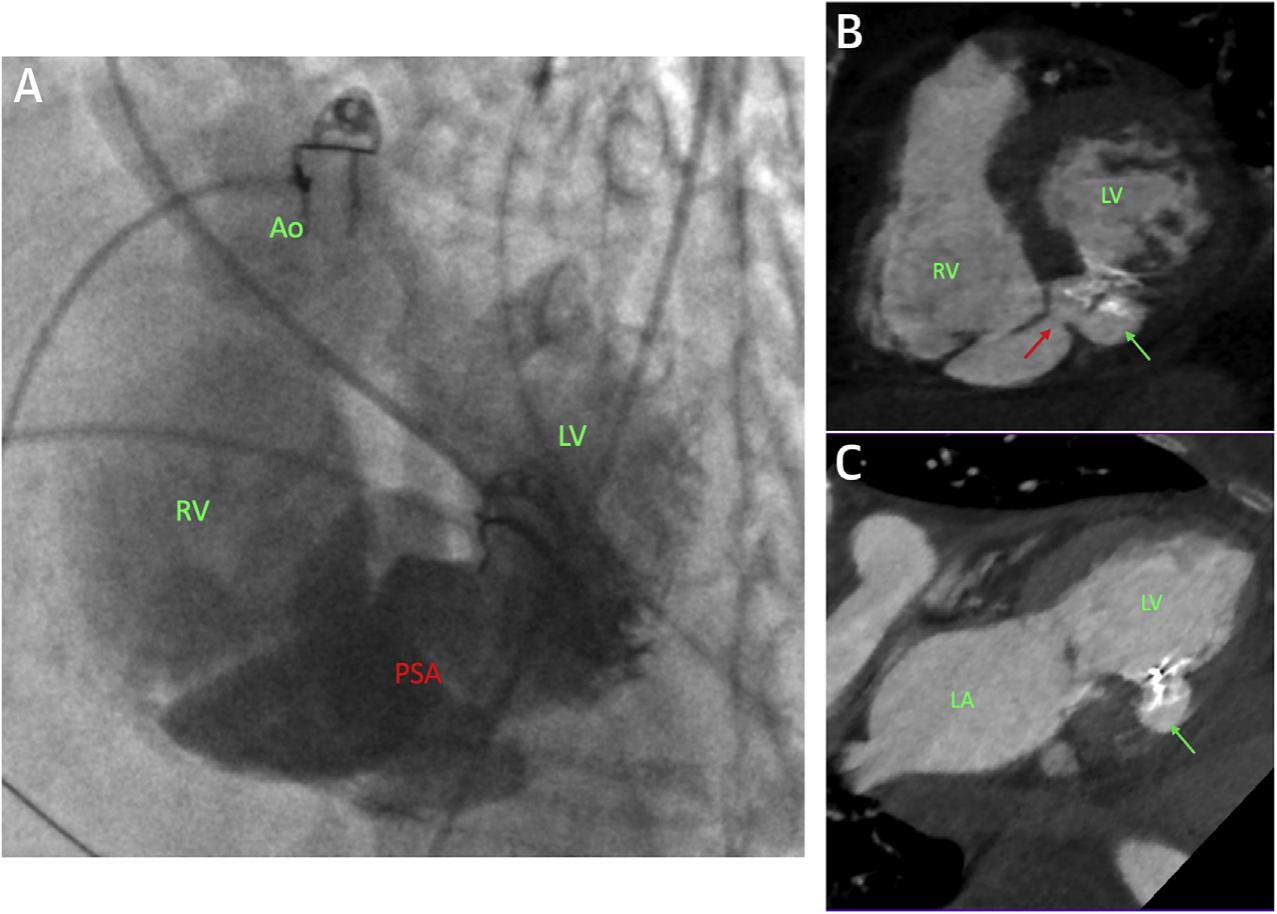
Biventricular Rupture With Communicating Pseudoaneurysm **(A)** Left ventricular (LV) angiogram from a left anterior oblique-cranial view showing a loculated pseudoaneurysm (PSA) arising from the septum and contrast in the right ventricle (RV). **(B and C)** Modified short-axis and 2-chamber views of the heart. The in situ occluder across the left ventricular defect and the pseudoaneurysm is seen **(green arrow).** The residual communicating right ventricular defect is also seen **(red arrow).** Ao = aorta; LA = left atrium.

Two weeks after her initial presentation, the patient underwent percutaneous repair using general anesthesia with TEE and fluoroscopy. LV angiography demonstrated a pseudoaneurysm; however, there was also contrast flow into the right ventricle (Figure 3A, Videos 5 and 6). Left femoral arterial and right internal jugular venous access was gained. The aortic valve was crossed in retrograde fashion with a 6-F JR4 catheter and a 0.035-inch hydrophilic wire. The LV defect was then crossed, and the wire was exchanged for a 300-cm Amplatzer Noodle wire (Abbott). This wire was snared in the pulmonary trunk from the venous side and externalized, thus creating an arteriovenous loop that provided favorable support for device delivery. The delivery catheter was advanced from the venous side and a 14-mm Amplatzer VSD occluder (Abbott) was deployed across the LV defect with no residual flow seen on TEE.

The patient recovered well. A CT scan was performed after her renal function improved (Figures 3B and 3C). This imaging confirmed rupture of the inferobasal left ventricle and a pseudoaneurysm with a well-positioned VSD occluder. There was also a small rupture of the inferior right ventricular (RV) wall communicating with the pseudoaneurysm. However, because the sac was now low pressure and contained, it was conservatively managed. The patient remains well >12 months later.

This is an unusual case of acute post-infarct biventricular rupture with a communicating pseudoaneurysm. It highlights the feasibility of percutaneous repair and importantly demonstrates that a small RV rupture into a low-pressure pseudoaneurysm may be conservatively managed with a good outcome.

## Conclusions

In appropriate patients, percutaneous repair of ventricular ruptures and pseudoaneurysms can be a viable minimally invasive option. Multimodality imaging and an MDT approach are crucial to maximize the chance of a durable result and a good clinical outcome.

## Figures and Tables

**Figure:**
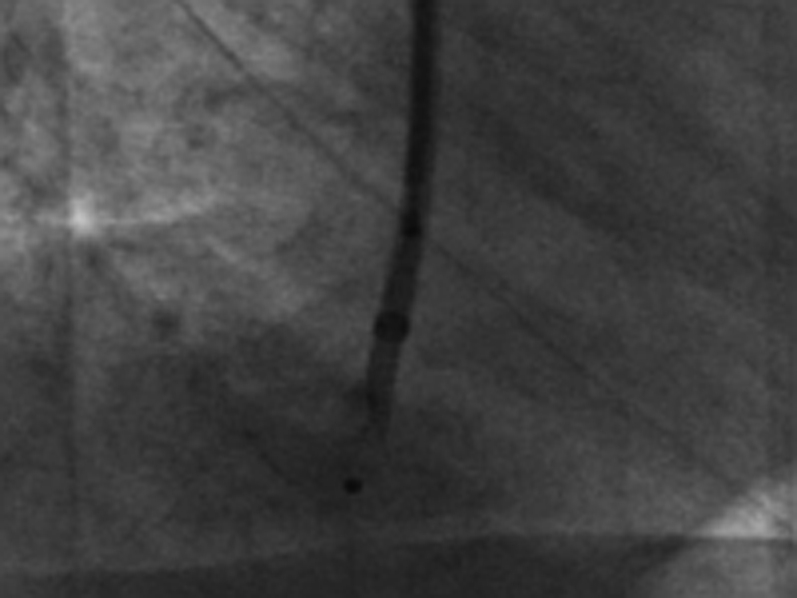
Online Video 1 Case 1 partial device deployment. Contrast injection into the left ventricle with the distal occluder disc deployed.

**Figure:**
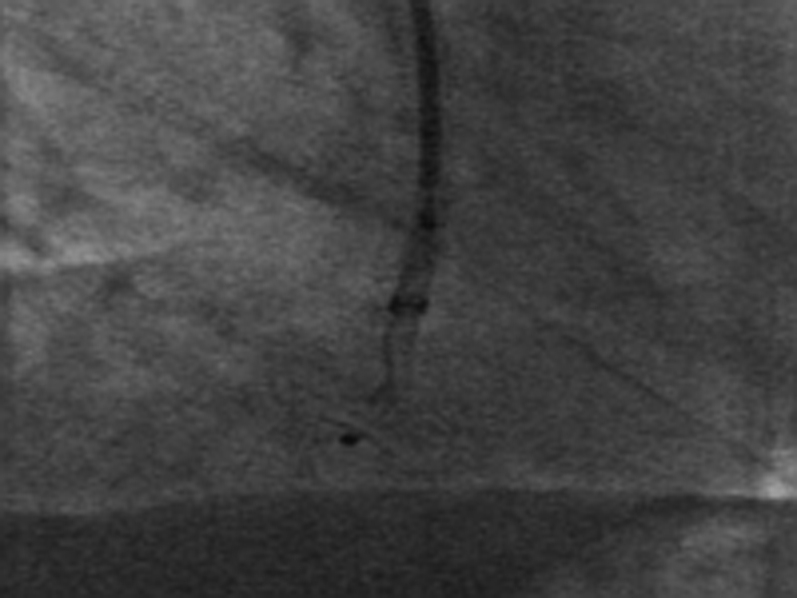
Online Video 2 Case 1 full device deployment. Deployment of the proximal occluder disc in the left ventricle.

**Figure:**
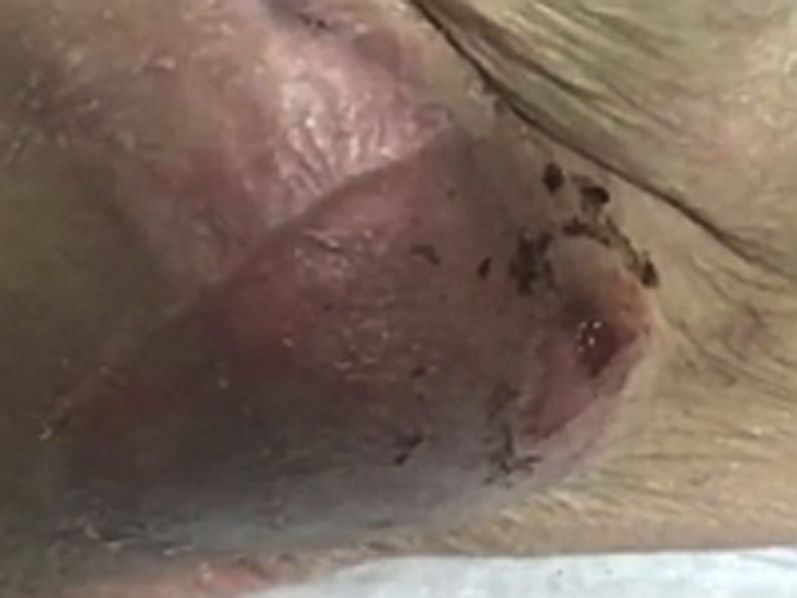
Online Video 3 Case 2 chest wall mass. Pulsatile chest wall mass caused by left ventricular apical pseudoaneurysm.

**Figure:**
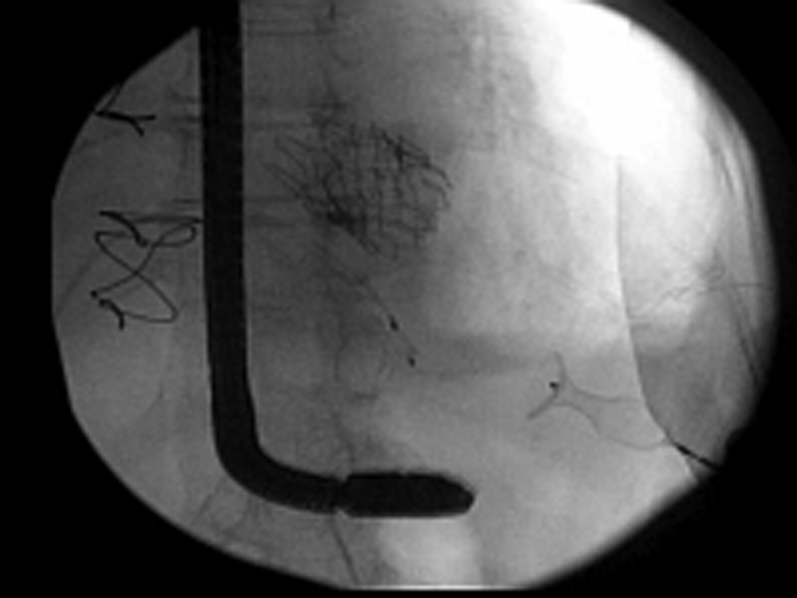
Online Video 4 Case 2 device deployment. Deployment of the occluder across the left ventricular rupture site.

**Figure:**
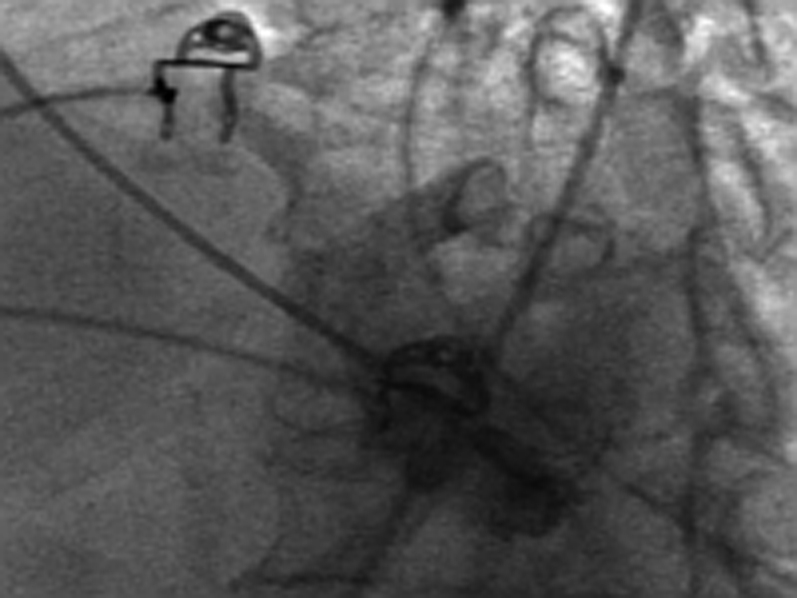
Online Video 5 Case 3 left ventricular angiogram (1). Left ventricular angiogram from a left anterior oblique-cranial view demonstrating the biventricular rupture with communicating pseudoaneurysm.

**Figure:**
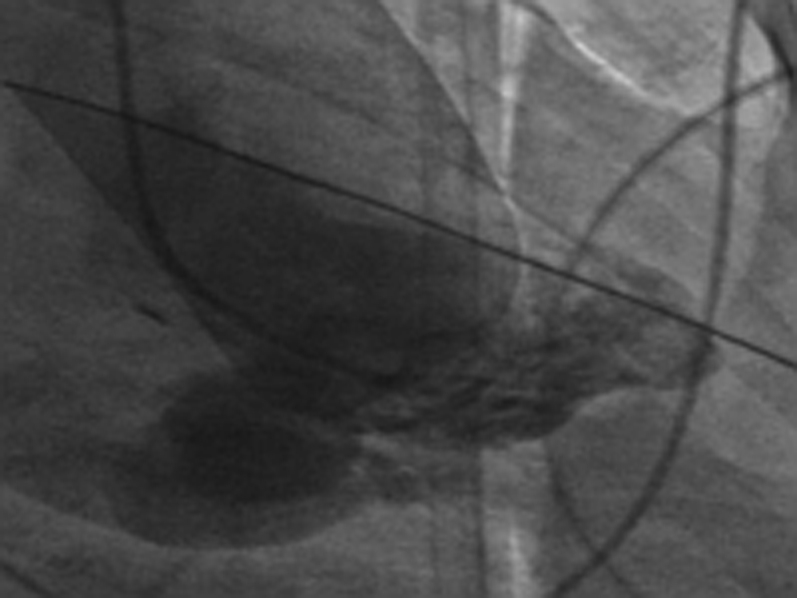
Online Video 6 Case 3 left ventricular angiogram (2). Left ventricular angiogram from an anterior-posterior projection.
